# Ionic Liquids Based on the Concept of Melting Point Lowering Due to Ethoxylation

**DOI:** 10.3390/molecules26134034

**Published:** 2021-07-01

**Authors:** Manuel Rothe, Eva Müller, Patrick Denk, Werner Kunz

**Affiliations:** Institute of Physical and Theoretical Chemistry, Faculty of Chemistry and Pharmacy, University of Regensburg, D-93040 Regensburg, Germany; Manuel.Rothe@ur.de (M.R.); Eva.Mueller@ur.de (E.M.); Patrick.Denk@ur.de (P.D.)

**Keywords:** ionic liquids, surface active ionic liquids (SAILs), room temperature ionic liquids (RTILs), alkyl ethylene glycol ether carboxylates, rare earth metals, transition metals, colloids, green chemistry

## Abstract

Most of the commonly used Ionic Liquids (ILs) contain bulky organic cations with suitable anions. With our COMPLET (Concept of Melting Point Lowering due to Ethoxylation), we follow a different approach. We use simple, low-toxic, cheap, and commercially available anions of the type C_x_(EO)_y_CH_2_COO^–^ to liquefy presumably any simple metal ion, independently of its charge. In the simplest case, the cation can be sodium or lithium, but synthesis of Ionic Liquids is also possible with cations of higher valences such as transition or rare earth metals. Anions with longer alkyl chains are surface active and form surface active ionic liquids (SAILs), which combine properties of ionic and nonionic surfactants at room temperature. They show significant structuring even in their pure state, i.e., in the absence of water or any other added solvent. This approach offers new application domains that go far beyond the common real or hypothetical use of classical Ionic Liquids. Possible applications include the separation of rare earth metals, the use as interesting media for metal catalysis, or the synthesis of completely new materials (for example, in analogy to metal organic frameworks).

## 1. Introduction

Some years ago, one of the authors of the present paper coauthored a publication entitled *The hype with Ionic Liquids as solvents* [1]. Indeed, sometimes it seems that this topic is (ab-)used as a machinery to increase the number of publications and the researchers’ impact factors rather than providing useful information about basic concepts or potential applications. 

Today, the majority of colleagues agree that Ionic Liquids are not necessarily “green”. Their synthesis usually is “red” and involves many steps and their lacking vapor pressure is not always a favourable property, because they cannot be separated by distillation. Further, they are often quite toxic, badly biodegradable, etc. 

However, they also undoubtedly have advantages. They enormously widen the spectrum of available liquids at room temperature. Although there is a general tendency to increasingly limit the number of industrially used solvents and liquids, ILs may provide alternatives to currently applied and well-known ones. In this sense, their role as “designer solvents” may be justified, provided that it is rigorously checked that existing classical solvents, which are usually cheaper, cannot fulfil equally well the intended purpose. 

So why do we need ILs? 

First of all, and almost trivial: they provide the opportunity to dissolve certain solutes better than conventional solvents, often by means of special solute–solvent interactions. Thus, a) the liquid state matters and b) the solubility performance is important. Compared to these features, the fact that the liquid consists of ions and therefore electrostatic interactions may play a prominent role, is (often) of minor importance, except, e.g., in electrochemistry. 

Second, and sometimes important and useful: the liquid may be structured in a way that goes beyond structuring in conventional low-molecular weight liquids. For example, there are surface-active, amphiphilic ILs (SAILs), where the imidazolium ring is connected to one or two long alkyl-chains thus creating cationic surfactants with convenient, usually small negatively charged counterions, mostly halides. Whereas these molecules may have some relevance when diluted in water (where they have some interesting properties, because of the smearing of the positive charges throughout the imidazolium ring), see [2] and references there, the current application in its pure IL state is rather limited. 

In light of the demands and the quest for new room-temperature liquids and solvents, it seems evident to look for alternative approaches to ILs that avoid the inherent shortcomings, such as laborious synthesis and purification and the limitation that always more or less complex organic cations are required. These facts motivated us to follow a different strategy: instead of lowering the melting points of salts via the bulkiness of the *cations* with, as a consequence, an increase of the solid-state Gibbs energy due to hindered packing, we lower the Gibbs energy of the liquid state by increasing the entropy of the *anions* [3]. It is, in fact, an already established concept and partly explains the relative success of the so-called Akypo™ surfactants, commercialized by the Japanese company Kao Chemicals. Note that the concept can also be applied to cations, but this is not part of the present paper [4]. 

The general chemical structure of the molecules is M^+^ C*_x_*EO_y_CH_2_COO^–^, which we call alkyl ethylene glycol ether carboxylates in what follows. Usually, these substances are commercialized as neutral acids, i.e., M^+^ = H^+^. Most interestingly, M^+^ can be any type of cation, even as simple as Na^+^, Li^+^, K^+^, NH_4_^+^, etc., but it can be also a complex cation and even a cationic surfactant. In the latter case, one can generate “true” cat-anionics, i.e., a mixture of cationic and anionic surfactants without any further counterions and this combination being even liquid at room temperature. 

Let us have a closer look at the anion. Evidently, if *x* ≥ 8, the anion is a surfactant, independently of *y*, but there are also very short anions available with *x* = 1–6 only. Then, the resulting ILs behave as hydrotropes or as “simple” more or less unstructured solvents (at least one would not expect any mesoscopic structuring). To have them be liquid at room temperature, a sufficient number of ethylene glycol groups (EO) is mandatory. These groups are not only significantly hydrated in water thus ensuring high water solubility, but they also exhibit a *high conformational entropy even in the pure state*. This is the secret behind their low melting points: they lose this entropy upon freezing, which is (Gibbs) energetically unfavorable. We called this approach to ILs *Concept of Melting Point Lowering due to Ethoxylation* (COMPLET) [3,5]. 

Compared to the “classical” concept to conceive ILs, several advantages are evident:

(i) The ingredients are readily available. Akypos are quite cheap and sold in tons. A simple mixing with alkali hydroxides and subsequent (freeze) drying is sufficient. No laborious synthesis and purification are necessary.
(ii) ILs can be made with simple cations and, as we demonstrate in what follows, even with di- and trivalent cations.
(iii) The ILs are of low toxicity and readily biodegradable so that they can be even used in cosmetics, of course, only with a convenient cation.
(iv) The anion in the IL can be surface active (and thus structuring) and can be liquid even with a surface-active cation forming “true cat-anionics” without further counterions.
(v) Some of them are even of very low viscosity at room temperature.

In the following sections, we discuss three different cases. In the first case, we consider ILs with metal cations of different valency, in the second case, surface active ILs based on the COMPLET concept, and finally, a particular case, where a partly protonated IL consists of direct micelles even in its pure state.

## 2. Ionic Liquids with Metal Ions of Different Valency

Ionic Liquids are often praised for their flexibility and customizability, to the point of being called “designer solvents”. In reality, however, this is only true to a degree. While a variety of properties can be achieved with conventional ILs, their makeup, and especially the strategy behind them, is often quite limited. Only a few choice cations act as the “heart” of nearly every “modern” Ionic Liquid, modified to suit the desired application. Besides some phosphonium- and ammonium-based ILs, the majority possesses an imidazolium cation as their “builder ion”. These ions can be modified and customized to a high degree and reliably lead to compounds with sufficiently low melting points to be classified as ionic liquids.

If one desires to incorporate metal ions as part of their ionic liquids, however, a problem arises: metal ions also carry positive charges and are thus inherently incompatible with all conventional “builder ions”. Naturally, there are established ways to circumvent this issue. The metal cations can be incorporated into larger complexes with a single negative excess charge, which is then compensated by one of the conventional “builder ions”. This strategy is often employed in the context of transition metals, which usually prefer higher oxidation states. A second common solution to the incompatibility issue is to skip the additional complexation compounds, and instead to attach residues to the “builder ion” that can bind to the metal ions [6,7,8]. 

Some arguments can be made against these two strategies. The transformation into a negatively charged complex adds additional compounds into the mix. They can alter the properties of the metal, for example, in catalytic applications, or introduce unwanted ions into the IL. Complexation by a sidechain of the main “builder ion” may allow the direct incorporation of bare metal ions, avoiding this issue. As the IL exists independently of the metal ion, however, one can also argue that instead of forming an ionic liquid with that metal ion, an IL is made that can only solubilize it instead. 

Ideally, one would combine an anionic builder ion directly with the bare metal cation. Although avoiding the aforementioned issues, another is introduced: many metal ions carry more than a single charge. The reason why this is an issue lies once again in the conventional concept for ionic liquids: they usually share a 1:1 stoichiometry. While ionic liquids with higher charges do exist, they are usually achieved by the simple linkage of several “builder ions” [7,8].

**Figure 1:**
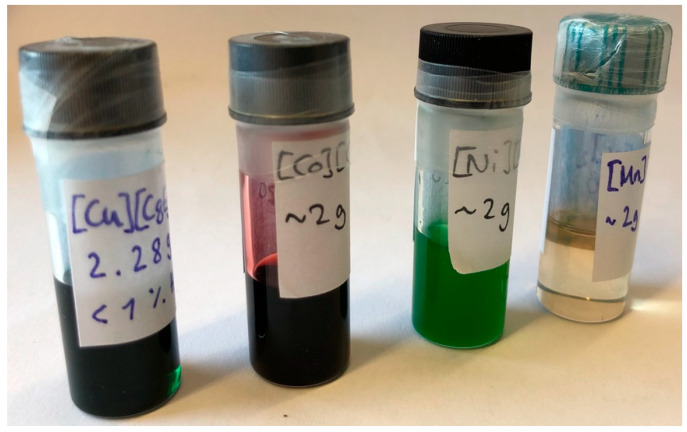
Typical COMPLET-based ionic liquids containing divalent transition metal ions.

With our alkyl ethylene glycol ether carboxylate salts, all these issues can be avoided. As they are built on a different concept, relying on entropy differences of the liquid and solid phases instead of enthalpy differences, they are much more flexible, especially when it comes to the combination with different cations. With C_8_EO_5_CH_2_COO^–^ ([C8E5c]), we put an anion at the center of the ionic liquid, and can easily combine it with a variety of metal ions. The COMPLET also allows for a deviation of the classical 1:1 stoichiometry, and di- and trivalent transition and rare earth metals ([REM]) can be easily incorporated (M = Fe^2+^, Fe^3+^, Cu^2+^, Co^2+^, Ni^2+^, Mn^2+^, Y^3+^, La^3+^, Dy^3+^, Eu^3+^, Gd^3+^). The synthesis of these ionic liquids is as simple as it can be: the free acid [H][C8E5c] is mixed with a basic metal salt, usually a hydroxide or carbonate, and stirred for several hours under gentle heating. Figure 1 shows a selection of typical divalent Ionic Liquids based on the COMPLET (M^2+^ = Cu^2+^, Co^2+^, Ni^2+^, Mn^2+^).

The resulting ILs show very diverse properties and ion-specific effects. As an example, the dynamic viscosities at 15 °C range from low, in the case of [Cu][C8E5c]_2_ (849 mPa s), to high, in the cases of [Eu][C8E5c]_3_ (126.6 Pa s) or [La][C8E5c]_3_ (152.2 Pa s), and very high, in the case of [Mn][C8E5c]_2_ (429.3 Pa s) [9]. The viscosity of a liquid is a measure of the mobility of its molecules, and as such it can provide some basic information about how the ILs may be structured. If the viscosity is high, the molecules within the liquid cannot freely flow past one another, and it is reasonable to assume that multiple metal centers are crosslinked via the alkyl ethylene glycol ether carboxylates.

To better understand the mobility within the liquid, specifically the ion mobility, viscosity measurements can be supplemented with the measurement of electrical conductivities. The combination of these two mobilities, so the general molecular mobility is given by the viscosity and the ion mobility is given by the electrical conductivity, provides information about the association of the ions in the IL. In an ideal case with full dissociation of the anion(s) and the cation, the system behaves like a strong electrolyte. In a double logarithmic plot of the molar conductivity Λ against the fluidity η^−1^, also called a *Walden Plot*, this behavior is represented by a line of unit slope. Such ILs are also often termed “good” ionic liquids. In contrast, “poor” ionic liquids show large negative deviations from this ideal line (often also “KCl line”) as a result of ion pairing [10,11]. It is important to keep in mind that this classification into “good” or “poor” does not imply the quality of the substance, but rather is a measure of its ionicity—a spectrum from fully ionic to molecular liquids. 

**Figure 2:**
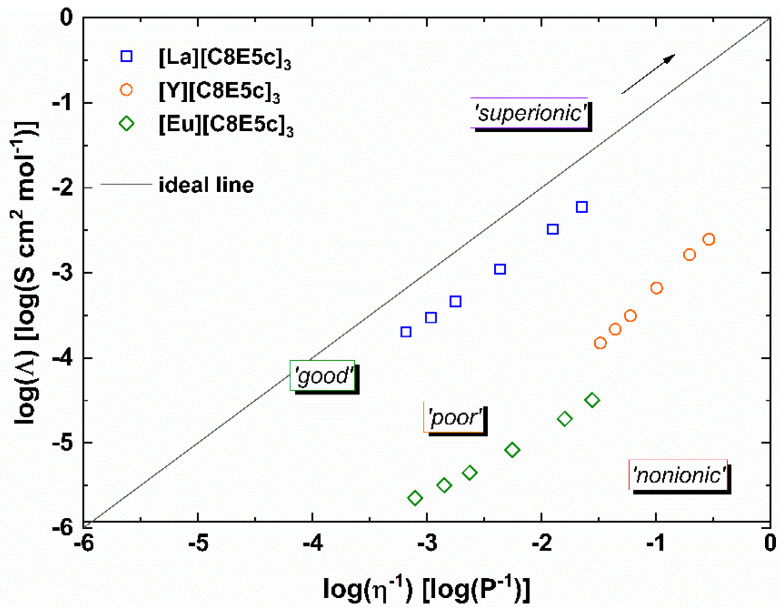
Walden Plot of selected rare earth metal ionic liquids in a range of 15–60 °C in comparison to the ideal KCl line, adapted from Reference [9] (Copyright © 2021 Wiley–VCH GmbH, Weinheim).

For three of these ionic liquids, a Walden Plot such as this has been previously created and can be seen in Figure 2 [9]. The ILs chosen are all based on rare earth metals, which are generally known to have quite similar properties. These similarities do not hold true in the case of our ILs. Not only do they possess vastly different fluidities, but also vary quite strongly in their measures of ion association. Both [Eu][C8E5c]_3_ and [Y][C8E5c]_3_ show significant negative deviations from the ideal line, indicating quite strong association of the ions. [La][C8E5c]_3_, however, remarkably displays only small deviations, and consequently, strong dissociation of the ions. This difference in ion mobilities and association presumptively is rooted in structural differences within the IL. It is known that related ionic liquids with alkali metals form both interconnected networks and globular complexes for sodium and potassium, respectively [12]. This difference in structure is also reflected in the difference in melting points—the sodium salt melts at −57 °C and the potassium salt at +60 °C [13]. Transition metals, and especially rare earth metals, have quite versatile complexation behavior and allow for much more diverse structures.

The diversity in properties does not end in these transport properties, but extends into other attributes with more direct application as well. We have previously shown that the solubilities of [REM][C8E5c]_3_ ILs in different solvents, or rather the resulting octanol/water partition coefficients *P*, are highly dependent on the associated cation, see Figure 2 [9]. These proof-of-concept separation experiments have already shown that the otherwise resource- and labor-intensive separation of rare earth metals is accessible with the use of alkyl ethylene glycol ether carboxylates. The partition coefficients are greatly affected by the presence of other ions, providing a highly sensitive and tunable system. The unique structure of the [C8E5c] anion also makes it responsive to changes in pH, due to its carboxylic acid, and changes in temperature, due to its ethylene glycol chain. 

This novel class of ionic liquids shows great promise in a multitude of applications due to their unique approach to liquefying metal ions. In combination with suitable metals, alkyl ethylene glycol ether carboxylate ionic liquids could be used as fluorescent dyes, in synthesis and catalysis, or as precursors for structured ceramics or nanoparticles. It is clear that the properties of the ionic liquids strongly depend on the choice of cation, and consequently, their application. However, the influence of the anion should not be forgotten: it is indubitably responsible for much of the solution and structuring behavior, and has also been shown to greatly affect fluorescence, enabling fluorescence in the visible spectrum for otherwise nonluminescent metal ions [9]. Variation and modification of the anion will surely enable the production of tailor-made, metal-based ionic liquids.

## 3. Surface Active Alkyl Ethylene Glycol Ether Carboxylates

In Reference [3], we have shown that the COMPLET concept can be used to liquefy cationic surfactants such as long-chain alkylammonium ones by combining them with short-chain methyl tetra ethylene glycol ether carboxylates. How to directly incorporate ethylene glycol groups into *cationic* surfactants to liquefy them with any type of anion, will be subject of several forthcoming papers, e.g., [4]. 

**Figure 3:**
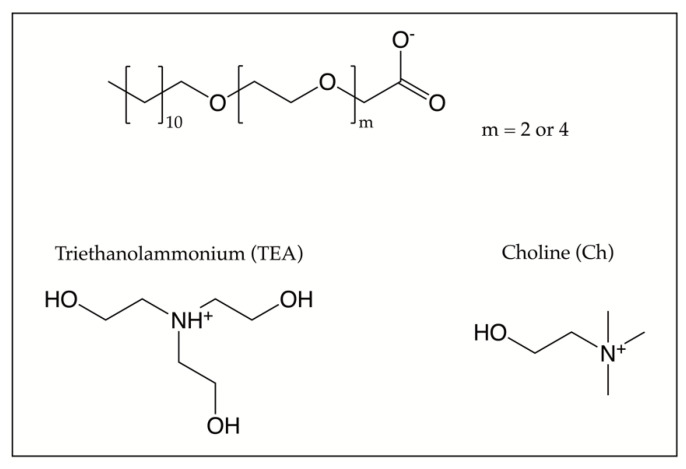
The anions and cations giving SAILs, as discussed in Section 3.

By contrast, in the present section, we focus on surfactants of the type dodecyl ethylene glycol ether carboxylate with two or four ethylene glycol groups ([C12E2c] or [C12E4c]) and either choline (Ch) or triethanolammonium (TEA) as counterions, see Figure 3.

Thermal stability is an important feature of Ionic Liquids. Commonly used organic solvents are characterized by either a high vapor pressure or a low boiling point, or both. Since Ionic Liquids do not have an appreciable vapor pressure, they can be applied in liquid state up to the temperature of thermal decomposition [8].

Therefore, thermal stability of the SAILs discussed here was studied using a thermogravimetric analyzer TGA 7 from Perkin-Elmer (Waltham, MA, USA). Samples were measured at a heating rate of 10 °C min^−1^, applying a continuous nitrogen flow. Decomposition temperatures were determined using onset points of mass loss, defined as the intersection of the baseline before decomposition and the tangent to the mass loss versus temperature.

**Figure 4:**
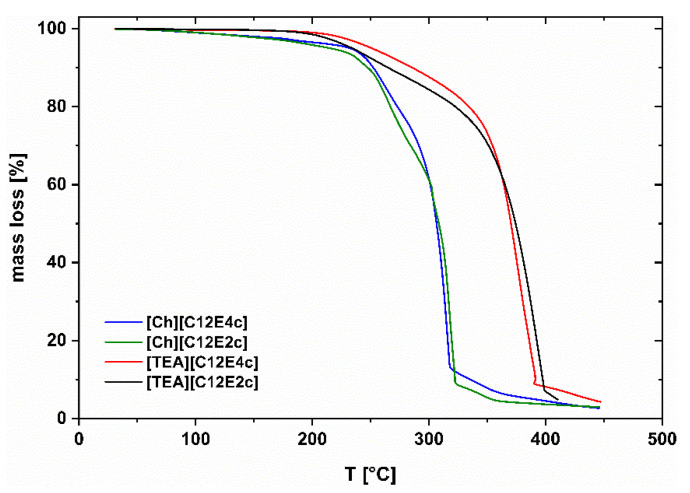
Temperature-dependent mass loss curves. Comparison of different counterions and degree of ethoxylation, adapted from Reference [15].

All the compounds tested show decomposition only above 200 °C. Decomposition temperatures of common Ionic Liquids can be found in the same range [14]. In the case of the pure substances, while the onset of decomposition is at slightly higher temperatures for Ch ILs, the TEA ILs exhibit higher bulk decomposition temperatures. Bulk decomposition temperatures are determined as the intersection between the tangent to the initial mass loss and the tangent to the more rapid mass loss at higher temperatures. No significant difference in thermal destruction occurs for Ch or TEA species with either two or four ethoxy units in the anionic part, see Figure 4. 

As can be seen in Figure 4, TEA leads to more resistant derivatives, while the point of decomposition decreases by 40 °C for choline species. Table 1 contains additional data for the four SAILs presented here. Further details about the experimental methods and the characterization of more SAILs can be found in [15]. Note that all of the SAILs have glass transition temperatures far below 0 °C, exhibit high viscosities at room temperature, and, for anionic surfactants, show relatively low critical micelle concentrations (cmc) when dissolved in water. They all exhibit a pseudoplastic behavior at room temperature. 

Unlike simple anionic surfactants, these SAILs usually only have a low tendency to form viscous liquid crystalline phases and are liquid at high concentrations and in absence of water.

## 4. The Special Case of M^+^ C_8_EO_8_CH_2_COO^−^

With x = 8 and y = 8, octyl octaethylene glycol ether carboxylic acid (C_8_EO_8_CH_2_COOH) with its commercial name Akypo LF2, is a surfactant consisting of a rather small hydrophobic and a much larger hydrophilic moiety. The comparatively long ethylene glycol chain ensures a sufficiently low melting point to keep the surfactant in its liquid state at room temperature, even in absence of water. As demonstrated above, C_8_EO_5_CH_2_COOH ([H][C8E5c]) (Section 2) and similar molecules (Section 3) can be deprotonated and combined with any metal cation, while retaining their liquid state, rendering them room temperature ionic liquids. Unsurprisingly, Akypo LF2 ([H][C8E8c]) can be treated similarly to obtain ionic liquids. 

In a recent paper [16], we reported on the aqueous phase behavior of [H][C8E8c] and elucidated its microstructuring over a wide range of concentrations by small-angle X-ray scattering (SAXS). Within the scope of that work, we also analyzed the effect of CaCl_2_ and NaOH on the phase behavior. Apart from a small coacervation regime at very low surfactant concentrations (<1 wt.%), the acid form features a phase behavior similar to that of the nonionic surfactant C_8_EO_8_ [17] with a typical clouding phenomenon. The lower critical solution temperature (LCST) of [H][C8E8c] was found to be 66 °C, thus significantly lower than the LCST of 96 °C reported for C_8_EO_8_. This difference can be attributed to enhanced intermicellar interactions, enabled by the additional carboxylic acid moieties. Ca^2+^ ions can act as intermicellar bridging agents between two carboxylate functions, as such lowering the LCST to 49 °C. As opposed to that, deprotonation of [H][C8E8c] with NaOH enhances its ionic character, completely suppressing the clouding phenomenon for [Na]_0.5_[H]_0.5_[C8E8c]. Yet, the phase behavior is remarkably simple in all cases. No liquid crystalline or reversed phases are observed, and the systems are isotropic, micellar liquids of relatively low viscosity at all ratios with water and all temperatures (above the critical micelle concentration and below the cloud point).

**Figure 5:**
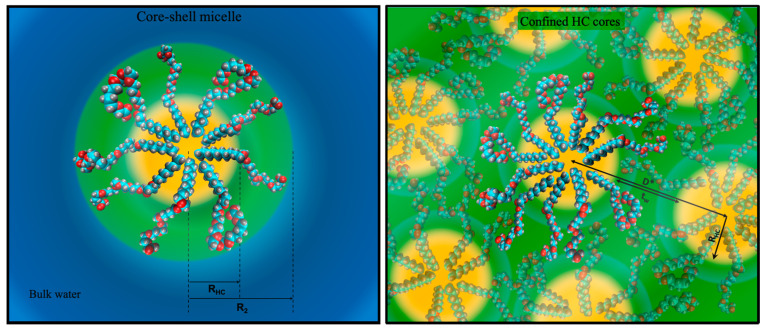
Scaled sketch of the two regimes observed in binary [H][C8E8c] water solutions. Left: the common core-shell structuring of direct micelles: a hydrocarbon core (R_HC_ = 1.2 nm, shown in yellow) is surrounded by a hydrated ethylene glycol shell (shown in green); the bulk represented in blue contains monomers at cmc. Right: the confined hydrocarbon core (HC) regime observed in water-poor solution with interdigitated headgroups and the absence of bulk water; in this regime, the interparticle distance gives a scattering peak at D* = 2π/q_max_. As can be inferred from osmotic pressure measurements, the compression of the water-poor ethylene glycol layer (t_w_) is responsible of the stability of the structure. The figure was reused from Reference [16] (Copyright © 2021 Elsevier Inc.).

A careful SAXS study revealed that [H][C8E8c] forms direct spherical micelles of constant size (hydrocarbon core radius R_HC_ ≈ 1.2 nm, aggregation number N_agg_ ≈ 30) and shape over the whole concentration range. Only at concentrations above 70 wt.%, when there is no longer any bulk water due to hydration of EO-groups, does the size start to decrease gradually. In the almost water-free state, N_agg_ is reduced to 8 and R_HC_ is reduced to approximately 0.8 nm. Obviously, the normal core-shell micelles cannot persist without any bulk water. Spherical micelles are then only possible, if the hydrophilic headgroups interdigitate and form a hydrophilic medium of more or less hydrated headgroups, in which the hydrophobic cores, consisting of hydrocarbon chains, are dispersed. Indeed, we could substantiate this idea through a combination of SAXS and vapor pressure osmometry measurements. Figure 5 is a sketch of the observed structures at high and low water concentrations. 

The effects of CaCl_2_ addition and transformation of [H][C8E8c] to [Na]_0.5_[H]_0.5_[C8E8c] (pH = pKa ≈ 4) can be summarized as follows. Neither the addition of CaCl_2_ nor the addition of NaOH changes the general structuring of the system. However, both partially suppress interdigitation and decrease the area per molecule a. In this case, a reduction of a corresponds to an increase of N_agg_. Yet, the mechanism leading to the partial suppression of interdigitation is different in both cases. Ca^2+^ ions can bridge carboxylate functions between or within micelles, inhibiting interdigitation either way. Nevertheless, Ca^2+^ enhances micellar attraction. Deprotonation of [H][C8E8c] with NaOH, on the other hand, enhances micellar repulsion by increasing the charge, consequently impeding headgroup interdigitation at the same time.

Since the publication of our referenced work [16], [Na][C8E8c] and [Ca][C8E8c]_2_ were prepared with less than 0.5 wt.% residual water. Both are isotropic ILs above their glass transition point. [Na][C8E8c] is liquid above 27.5 °C, whereas [Ca][C8E8c]_2_ is already liquid above 19.5 °C. These ionic liquids will be investigated by SAXS in the future, but extrapolation of our published data suggests that they also consist of spherical hydrocarbon cores, dispersed in a hydrophilic medium of interdigitated headgroups. If this is the case, these ionic liquids are highly interesting, because they possess (a) hydrophobic nanodroplets dispersed in a hydrophilic medium with a large interface and (b) the carboxylate functions enable charge transfer between both domains, since the EO-chains are highly flexible. Owing to those features, the [C8E8c] SAILs seem to be very promising solvents for catalytic purposes. Nanoparticle synthesis in such a confined environment may also be conceivable.

**Figure 6:**
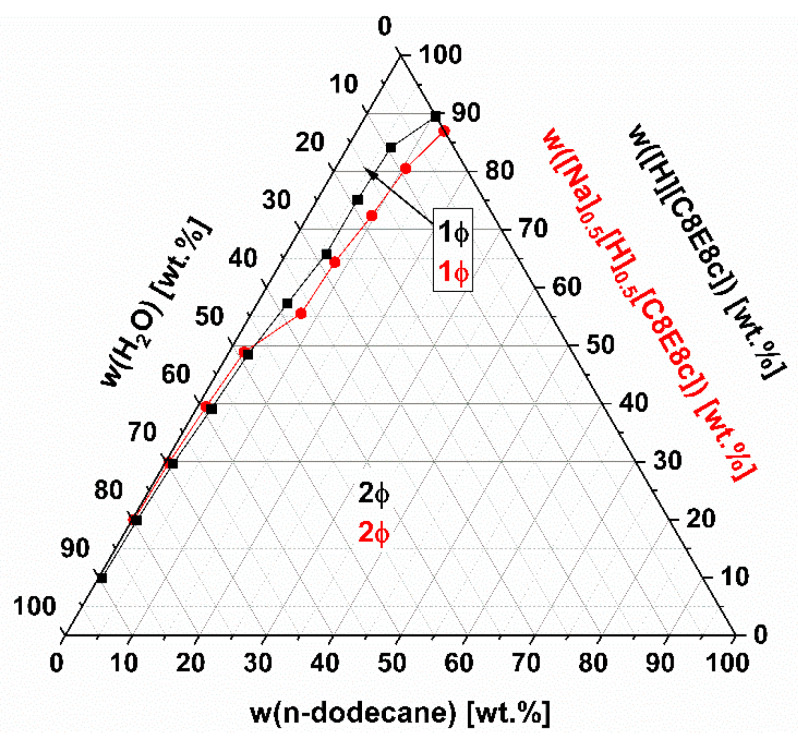
Ternary phase diagrams of [H][C8E8c]/H_2_O/n-dodecane (■) and [Na]_0.5_[H]_0.5_[C8E8c]/H_2_O /n-dodecane (●) at a temperature of 23 °C (±1 °C). Concentrations are given in wt.%. The precision in w(n-dodecane) is usually ±0.5 wt. %, whereas the deviation of the other weights is negligible; 1ϕ denotes a monophasic system, and 2ϕ, realms of existence of two liquids in equilibrium.

Concerning catalysis of organic reactions, it is also worth considering the phase behavior of the SAILs on the addition of hydrophobic solvents and water. Ternary phase diagrams of the systems [H][C8E8c]/H_2_O/n-dodecane and [Na]_0.5_[H]_0.5_[C8E8c]/H_2_O/n-dodecane at T ≈ 23 °C are given and compared in Figure 6. Note that the glass transition of dry [Na]_0.5_[H]_0.5_[C8E8c] (<0.5 wt.% H_2_O) is shifted to lower temperatures compared to that of neat [Na][C8E8c]. It is liquid at temperatures above approximately 22–23 °C. In both cases, at any surfactant concentration, only a certain amount of n-dodecane can be solubilized. Any excess n-dodecane phase separates. This can be explained by the incorporation of n-dodecane into the hydrophobic cores of the (interdigitated) micelles, which is only possible to a certain extent, as the packing of the surfactant molecules is constrained and most likely only allows spherical structures. Further, it is likely that the separating n-dodecane phase contains almost no surfactant due to its inability to form reversed structures. Since there is also a phase separation in the case of the water-free SAIL, unlike, for example, the case of the nonionic surfactant C_8_EO_6_, which is completely miscible with n-dodecane [18], a simple extraction of possible reaction products might be feasible. [H][C8E8c] can uptake about 10 wt.% n-dodecane, and [Na]_0.5_[H]_0.5_[C8E8c] up to 13 wt.%.

## 5. Conclusions and Outlook

Compared to classical ionic liquids (ILs), the ILs based on COMPLET (Concept of Melting Point Lowering due to Ethoxylation) offer numerous advantages. They are very easy to prepare, they are cheap, exhibit low toxicity, and the organic part is readily biodegradable when appropriate simple ions such as Na^+^, Fe^2+^, etc. are chosen. Further, they can be used to liquefy virtually any cation, be it mono-, di-, or triple-charged, at room temperature. Since the ILs presented here are liquefied via the high flexibility of the organic anions, simple cations such as Na^+^ and Li^+^ can be used as well as Fe^2+^ or Fe^3+^ or Eu^3+^ or, probably, any other cation that is interesting for catalysis or that must be extracted and recovered. The anions can also be surface-active. Although surface-active ILs (SAILs) are already known, the COMPLET-based SAILs offer anionic surfactants, which are much more relevant for large applications than cationic ones. They are available in large quantities, they are easily dilutable with water and hydrophobic solvents, depending on the alkyl-chain length, and the formation of highly viscous liquid crystals upon water dilution can be widely avoided. 

On the other hand, COMPLET-based ILs offer several advantages just as other ILs: they are of low volatility and liquid and stable over a wide temperature range. Finally, the properties, ranging from surfactant-like via hydrotrope-like to purely solvent-like, can be tuned at will by conveniently choosing the appropriate alkyl-chain length, typically C1 to C12, provided that the number of ethylene glycol groups is sufficient.

Given all these advantages, we believe that this type of ILs may have a significant future and impact on improved industrial processes and products. We invite researchers interested in Ionic Liquids to try these and to further deepen our knowledge on them.

## Figures and Tables

**Table 1:** Decomposition temperature, glass transition temperature, and viscosities at two temperatures for the four salts (SAILs) considered, together with their rheological behavior. For aqueous Scheme 15.

Composition	T Decomp. [°C]	T glass Trans. [°C]	Cmc [mol/L]	Viscosity 20 °C [Pa s]	Viscosity 80 °C [Pa s]	Rheology
**[TEA][C12E2c]**	222	−39.0	2 × 10^−4^	~100	~4	pseudoplastic
**[Ch][C12E2c]**	240	−44.0	2 × 10^−4^	~30	~5	pseudoplastic
**[TEA][C12E4c]**	234	−42.0	2 × 10^−4^	~60	30 × 10^−3^	pseudoplastic ≤ 70 °C Newtonian > 70 °C
**[Ch][C12E4c]**	241	−49.5	2 × 10^−4^	~80	10 × 10^−3^	pseudoplastic ≤ 60 °C Newtonian > 60 °C

## Data Availability

Additional data are available on request to the authors.

## Sample Availability

Samples of the compounds are not available from the authors.
